# Shensongyangxin Capsules for Paroxysmal Atrial Fibrillation: A Systematic Review of Randomized Clinical Trials

**DOI:** 10.1371/journal.pone.0151880

**Published:** 2016-03-21

**Authors:** Guang Chen, Benjun Wei, Jie Wang, Bo Feng, Zhaoling Li, Zhenpeng Zhang, Qingyong He

**Affiliations:** 1 Department of Cardiology, Guang’anmen Hospital, China Academy of Chinese Medical Sciences, Beixiange 5, Xicheng District, Beijing, 100053, China; 2 Beijing University of Chinese Medicine, Beijing, 100029, China; 3 Hubei University of Chinese Medicine, Hubei, 430065, China; Cardiff University, UNITED KINGDOM

## Abstract

**Objective:**

To evaluate the evidence for the effectiveness and safety of Shensongyangxin Capsules (SSYX) for treating paroxysmal atrial fibrillation (PAF).

**Methods:**

We searched for randomized clinical trials for SSYX in PAF up to June 2015. The Cochrane risk of bias tool was used to assess the methodological quality. RevMan 5.3 was used to synthesize the results.

**Results:**

We included 22 trials involving 2,347 PAF patients. The quality of the included studies was generally poor. The results of the meta-analysis showed that SSYX plus routine treatment was more effective at improving P-wave dispersion (Pwd) and the frequency of PAF attacks compared with routine treatment alone. The results from the included trials that compared SSYX plus routine treatment and arrhythmic drugs plus routine treatment were inconsistent. Trials reported on Pwd, quality of life, frequency of PAF attacks or maintenance rate of sinus rhythm and found that SSYX combined with anti-arrhythmic drugs plus routine treatment was more effective than anti-arrhythmic drugs plus routine treatment. Four of the trials reported adverse events, indicating that SSYX was potentially safer than anti-arrhythmic drugs.

**Conclusions:**

There appears to be some benefit from the use of SSYX. However, due to poor methodological quality, we could not draw confirmative conclusions regarding the beneficial effect of using SSYX.

## Introduction

Atrial fibrillation (AF) is the most common arrhythmia and the most serious disorder of atrial electric activity that is associated with the regular displacement of the P-wave by the atrial fibrillary wave. Epidemiological evidence from the USA, the UK, Japan and China suggests that AF is becoming more prevalent [1–5] and that its highest prevalence and incidence is observed in the 80-years-old and over group, with rates in males being higher than in females [1,3,5,6]. Patients who have AF frequently experience a range of comorbidities, which complicates treatment and affects long-term prognosis. Previous surveys have confirmed that AF increases annual total healthcare costs [7–11]. Paroxysmal atrial fibrillation (PAF) consistently tends to develop into persistent and permanent atrial fibrillation. Its main clinical manifestation is recurrent attacks of palpitations and chest distress. AF and heart failure (HF) often coexist, and this combination leads to severe symptoms. There is no evidence to support the idea that controlling the rhythm is better than controlling the heart rate in efforts aimed toward stroke prevention and reducing the death rate. The initial aim of treatment is therefore to reduce the symptoms of the attack. Catheter ablation and drug therapy are important parts of treatment for patients with PAF. Suggested drug therapy includes blood-clot prevention, controlling the ventricular rate, converting AF and maintaining sinus rhythm as well as treating indications, such as protopathy, and complications, such as hypertension, coronary heart disease (CHD), HF and hyperlipidemia. As for PAF, it is specifically important to maintain sinus rhythm, but the curative effects of western medicines on PAF are still unsatisfactory. Moreover, during long-term treatment with western medicines, typical side effects arise, including the occurrence of other malignant arrhythmias.

Shensongyangxin Capsules (SSYX) are a pure Chinese medicine composed of *Radix Ginseng Alba* (rén shēn), *Radix Ophiopogonis* (mài dōng), *Fructus Corni* (shān zhū yú), *Radix et Rhizoma Salviae Miltiorrhizae* (dān shēn), *Semen Ziziphi Spinosae* (suān zăo rén), *Herba Taxilli* (sāng jì shēng), *Radix Paeoniae Rubra* (chì sháo), *Eupolyphaga seu Steleophaga* (tŭ biē), *Radix et Rhizoma Nardostachyos* (gān sōng), *Rhizoma Coptidis* (huáng lián), *Fructus Schisandrae Sphenantherae* (nán wŭ wèi zĭ) and *Os Draconis* (lóng gŭ), that tonify Qi and nourish Yin and promote blood circulation to remove meridian obstruction to clear away heart fire and calm the mind. PAF manifests as palpitation, which is associated with patterns of Qi deficiency and heart blood stasis according to theories of traditional Chinese medicine (TCM). Therefore, treating PAF with SSYX and integrated traditional and western medicine has been an extensively covered topic over the last few decades. A flurry of clinical trials emerged in mainland China and many randomized controlled trails (RCTs) were published, but these have not been evaluated according to the PRISMA systematic review standards.

The pharmacological mechanism by which SSYX acts in treatments for PAF is still being explored. However, it is not rare in China for patients who suffer from recurrent PAF attacks to refer to SSYX because Chinese medicine is believed to work as a healing method for treating a range of disorders, especially blood stasis and Qi deficiency. Moreover, there is a belief that the adverse events that are attributed to anti-arrhythmic drugs might be prevented by SSYX.

The aim of this systematic review is to evaluate the evidence for the effectiveness and safety of SSYX compared to placebo and anti-arrhythmic drugs by analyzing the influence of attack times and P-wave dispersion of PAF.

## Methods

We undertook this meta-analysis of clinical trials in conformity with the guidelines put forth by the Preferred Reporting Items for Systematic Reviews and Meta-analyses (PRISMA) statement (S1 PRISMA Checklist), and we registered the protocol on the PROSPERO International prospective register of systematic reviews (No.CRD42015023968) (http://www.crd.york.ac.uk/PROSPERO/).

### Search strategy

The literature research was conducted using the PubMed database, the Cochrane Library, the China National Knowledge Infrastructure (CNKI) database, the China Scientific Journal Database (VIP), the Wanfang Database and the Chinese Biomedicine literature service system (SinoMed), covering the period from the earliest possible year up to June 2015. The following search terms (or the Chinese equivalent for Chinese databases) were used individually or cross-linked and varied depending on which database was being searched: “Shensongyangxin Jiaonang”, “Shensongyangxin Capsules”, “Shensongyangxin”, “atrial fibrillation”, “auricular fibrillation” and “random”.

The specific search strategy of CNKI was as follows:

#1 Search((atrial fibrillation [Subject])OR auricular fibrillation[Subject])
#2 Search(((Sensongyangxin Jiaonang[Subject]) OR Sensongyangxin Capsules[Subject]) OR Sensongyangxin[Subject])
#3 Search (random [Full Text])
#1 and #2 and #3

### Eligibility criteria

#### Types of trials

All randomized controlled trials (RCTs) investigating the use of SSYX in the treatment of PAF were eligible for inclusion.

#### Types of participants

All adult patients (18 years and older, no upper age limit) with a diagnosis of PAF were considered for this review. We followed the guidelines of the 2012 European Society of Cardiology (ESC) preliminary criteria for the clinical diagnosis of PAF and the 2014 AHA/ACC/HRS Guideline for the Management of Patients With Atrial Fibrillation [12, 13](Box 1).

**Box 1 Preliminary criteria for the clinical diagnosis of PAF from ESC Guidelines for the management of atrial fibrillation [12]**

AF was defined as a cardiac arrhythmia with the following characteristics:

1. The surface ECG showed ‘absolutely’ irregular RR intervals: RR intervals that did not follow a repetitive pattern.
2. There were no distinct P waves on the surface ECG. Some apparently regular atrial electrical activity may have been observed in some ECG leads, most often in lead V1.
3. The atrial cycle length (when visible), i.e., the interval between two atrial activations, was usually variable and <200 ms (>300 bpm).

Paroxysmal AF is self-terminating, usually within 48 h. Although AF paroxysms may continue for up to 7 days, the 48 h time point is clinically important because after this, the likelihood of spontaneous conversion is low, and anticoagulation must be considered.

#### Types of interventions

Shensongyangxin capsules, or routine treatment plus shensongyangxin, was compared with routine treatment, the administration of other anti-arrhythmia drugs, placebo or no intervention. The dose of shensongyangxin capsules was 1.2 g to 1.6 g in 3 to 4 capsules (0.4 g in each capsule) administered 3 times per day for a total dose between 3.6 g to 4.8 g per day.

#### Types of outcome measures

Primary outcome measures

1. P-wave dispersion (Pwd)
2. Maintenance of sinus rhythm

Secondary outcome measures

1. Quality of life, such as QOL Scale
2. Frequency of PAF attack
3. Symptom improvement (such as palpitations, chest distress, etc.)

### Study selection and data extraction

Two authors independently performed the literature searches, screening, and data extraction. The extracted data included the title of the study, the authors, the year of publication, the article source, the study size, the total number of cases, the percentage of cases with a male patient, the mean age of the patients, the setting of the patients, the diagnosis standard, the grouping details used for methodological information, and the treatment process in addition to details regarding control interventions, outcomes, and adverse effects for each study. Disagreements were resolved by discussion and were arbitrated by a third author.

### Assessment of risk of bias

Two authors (Chen G and Wei BJ) independently assessed the methodological quality of the included RCTs using RevMan 5.3.0, according to the criteria for judging risk of bias in the ‘Risk of bias’ assessment tool in the Cochrane Handbook [14]. The following items were assessed: random sequence generation (selection bias), allocation concealment (selection bias), blinding of participants and personnel (performance bias), blinding of outcome assessment (detection bias), incomplete outcome data (attrition bias), and selective outcome reporting (reporting bias). Sample size estimates, comparable baseline characteristics, and inclusion and exclusion criteria were considered as other biases. The quality of each of the included RCTs was categorized as having a low, unclear, or high risk of bias. Disagreements were resolved by discussion with a third author (Wang J).

### Data analysis

RevMan 5.3.0, provided by the Cochrane Collaboration, was used to analyze the results of the trials. The meta-analysis was performed if the patients, interventions, controls, and outcomes were the same or similar and the data were sufficiently homogeneous. Continuous outcomes were expressed as a weighted mean difference (WMD), and dichotomous data were expressed as relative risk (RR); 95% confidence intervals (CI) were calculated for both. In the absence of significant heterogeneity, we pooled data using a fixed effects model; otherwise, we used a random effects model [14]. Heterogeneity was assessed using both Chi-squared tests and the I-squared statistic with an I-squared value greater than 50% indicating substantial heterogeneity. Funnel plots were generated to detect publication bias when more than ten trials were identified. When the necessary data were available, subgroup analysis was performed for different parameters that represented the conditions of the PAF attacks. To minimize bias in our findings and recommendations, we used the Grading of Recommendations Assessment, Development, and Evaluation (GRADE) summary of findings table for outcomes to grade the available evidence.

## Results

### Study search and selection

**Fig 1:**
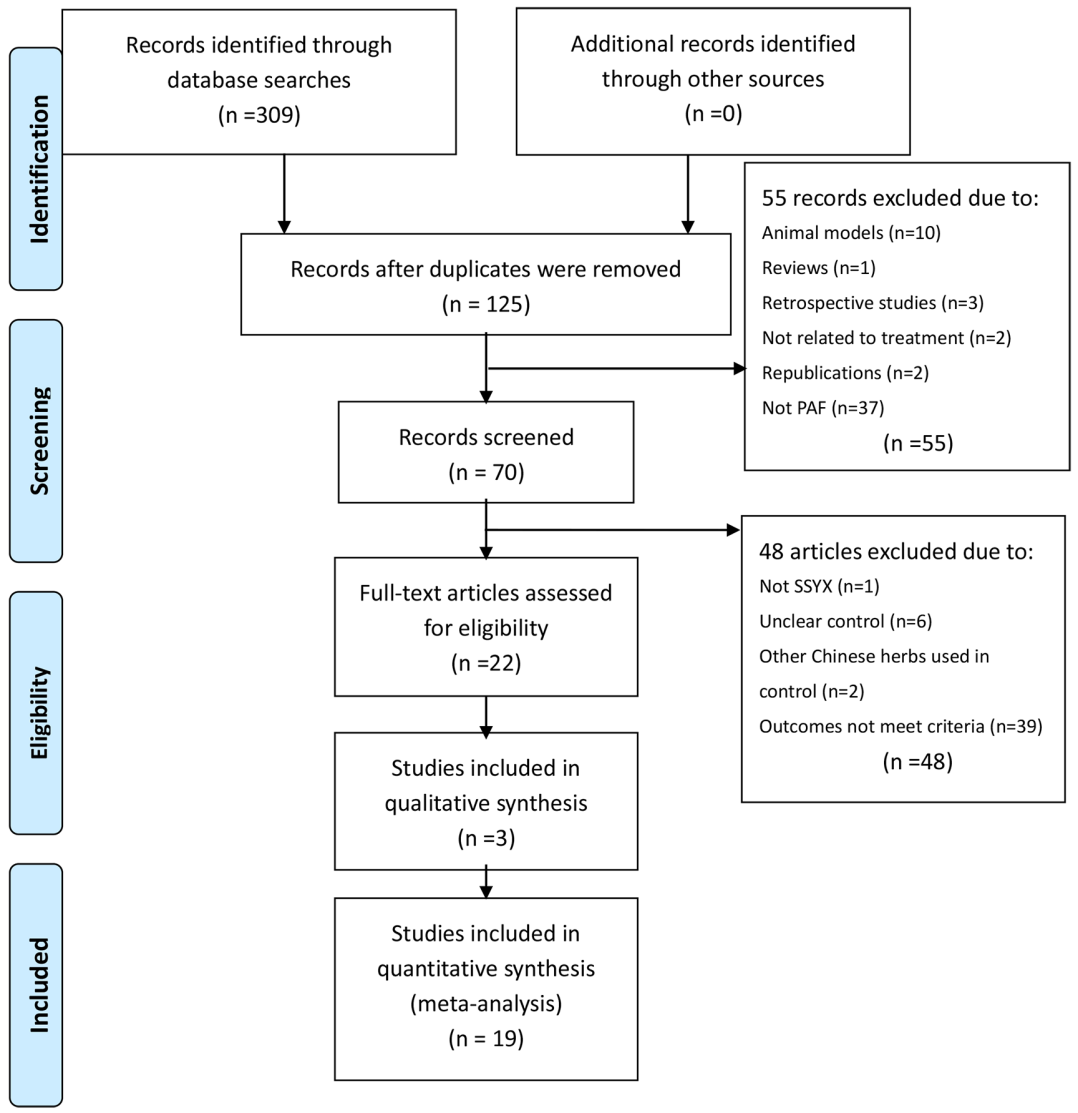
The selection process of this systematic review.

The initial search in June 2015 retrieved 287 articles that related to treatment for PAF from six databases. After duplicates were removed, 118 theses were identified, including 9 trials published in English and 109 published as Chinese journal articles. Following a review of the titles and abstracts of these publications, 55 studies were excluded, and only 63 studies remained. Two trials were excluded because of duplicated publication, ten trials were excluded for being animal studies, and six trials were excluded for being nonclinical trials, including retrospective studies and traditional reviews. In the end, after full text review, 41 out of the remaining 63 articles were excluded based on the inclusion criteria of intervention, control, and outcomes, which left 22 trials[15–36] to be included. The screening process is summarized in a flow chart (Fig 1).

### Study characteristics

The characteristics of the 22 RCTs are listed in Table 1. The 22 RCTs, which involved 2347 participants aged 18 to 79 years old, were published between 2007 and 2013. Sample sizes varied from 41 to 232 participants, with an average of 106.68 participants per trial. All trials declared that the included patients met diagnostic criteria for PAF but failed to describe the specific diagnostic criteria. There was only one multicenter trial[27]. All of the trials had two arms except five trials [20, 27,23,24,25] that had three arms. There were six trials that compared SSYX capsules plus routine treatment with routine treatment alone, eight trials that compared SSYX capsules plus routine treatment to anti-arrhythmia drugs plus routine treatment, and fourteen trials that compared SSYX combined with anti-arrhythmia drugs plus routine treatment to the same anti-arrhythmia drug plus routine treatment. The five trials with three arms had two comparisons, which we counted twice. SSXY capsules were patent medicines approved by the State Food and Drug Administration of China. The daily dosages of SSYX were 3.6 g in two studies [15, 35], 2.4 g to 4.8 g in two studies [29, 32] and 4.8 g in the other 19 studies. Treatment courses ranged from 8 to 24 weeks.

**Table 1:** Characteristics of patients, interventions, and outcomes that were compared in the studies included in this systematic review. NR, not reported; t, treatment group; c, control group; HBP, high blood pressure; CHD, coronary heart disease; DCM, dilated cardiomyopathy; RHD, rheumatic heart disease; DM, diabetes mellitus; HF, heart failure; LAD, left atrium canon; LVEF, left ventricular ejection fraction; CRP, C-reactive protein; IL, interleukin.

ID	Sample size (treatment/control)	mean age (years)	% men	complications	intervention	comparison	Treatment duration (weeks)	outcome measure
Shao JK 2013[15]	80 (40/40)	t: 67.7±5.2 c: 68.1±5.7	56.25%	NR	SSYX capsule 1.2 g tid + routine treatment, including rosuvastatin calcium 10 mg qd	no intervention (routine treatment only)	8	Frequency of PAF attack, effective rate
Han YH2010[16]	100 (50/50)	t: 59±12.6 c: 57±11.3	70.00%	HBP	SSYX capsule 1.6 g tid + routine treatment (specific drug was unclear)	no intervention (routine treatment only)	24	Frequency of PAF attack
Sheng HP 2010[17]	60 (30/30)	t: 55.7 c: 56.3	51.67%	HBP CHD RHD	SSYX capsule 1.6 g tid + routine treatment (specific drug was unclear)	no intervention (routine treatment only)	24	Frequency of PAF attack, Life quality
Lu JZ 2012[18]	114 (58/56)	t: 57.2±8.4 c: 56.3±8.3	43.86%	HBP CHD	SSYX capsule 1.6 g tid + routine treatment, including aspirin 100 mg qd + simvastatin 20 mg qn	no intervention (routine treatment only)	24	Pwd, hemorhelogical parameters, LAD, LVEF
Liu XC 2012[19]	46 (23/23)	51.00±11.32	NR	HBP CHD	SSYX capsule 1.6 g tid + routine treatment (specific drug was unclear)	no intervention (routine treatment only)	12	Pwd, hsCRP;IL-6
Quan T 2008[20]	41 (20/21)	NR	NR	NR	SSYX capsule 1.6 g tid + routine treatment (specific drug was unclear)	no intervention (routine treatment only)	12	Pwd, effective rate
Quan T 2008[20]	41 (20/21)	NR	NR	NR	SSYX capsule 1.6 g tid +routine treatment (specific drug was unclear)	Kredex carvedilol (10 mg qd first time and increased to 10 mg bid the next day and maintained) + routine treatment	12	Pwd; effective rate
Men R 2012[21]	84 (42/42)	t: 63.5±7.2 c: 64.6±6.9	75.00%	HBP CHD RHD	SSYX capsule 1.6 g tid + routine treatment (specific drug was unclear)	Amiodarone 0.2 g tid, decreased to bid a week later and decreased to qd the next week later and maintained at qd + routine treatment	12	Frequency of PAF attack; Life quality
Li L 2009[22]	85 (42/43)	NR	NR	NR	SSYX capsule 1.6 g tid + routine treatment (specific drug was unclear)	Amiodarone 0.2 g tid, decreased to bid a week later and decreased to qd the next week later and maintained at qd + routine treatment	24	Frequency of PAF attack
Liu YX 2013[23]	108 (52/56)	t: 75.4±16.1 c: 72.4±15.2	55.56%	CHD	SSYX capsule 1.6 g tid + routine treatment (specific drug was unclear)	Amiodarone 0.2 g tid, decreased to bid a week later and decreased to qd the next week later and maintained at qd + routine treatment	8	Pwd
Jin YW 2012[24]	43 (21/22)	t: 63.94±14.26 c: 66.03±14.17	72.09%	HBP CHD RHD DCM	SSYX capsule 1.2 g tid + routine treatment (specific drug was unclear)	Metoprolol 23.75 mg qd, increased to 47.5 mg qd according to tolerance capability of patients + routine treatment	12	Pwd; CRP
Wu PT 2007[25]	113(58/55)	t: 56±3 c: 58±5	49.62%	HBP CHD HF	SSYX capsule 1.6 g tid + routine treatment (specific drug was unclear)	Metoprolol (dosage is unclear) + routine treatment	12	Pwd
Li YX 2011[26]	80(40/40)	t: 56.4 c: 56.6	60.00%	HBP CHD RHD	SSYX capsule 1.6 g tid + routine treatment (specific drug was unclear)	Amiodarone 0.2 g tid, decreased to bid a week later and decreased to qd the next week later and maintained at qd + routine treatment	12	Pwd, symptom
Wang AH 2011[27]	232(117/115)	t: 58.0±12.0 c: 63.0±9.0	56.03%	HBP CHD DM HF	SSYX capsule 1.6 g tid + propafenone analogues +routine treatment (specific drug was unclear)	Propafenone analogues (150 mg, tid) + routine treatment	8	Frequency of PAF attack, effective rate, duration of PAF attack
Quan T 2008[20]	42(21/21)	NR	NR	NR	SSYX capsule 1.6 g tid + kredexcarvedilol (10 mg qd first time and increased to 10 mg bid the next day and maintained) + routine treatment (specific drug was unclear)	Kredexcarvedilol (10 mg qd first time and increased to 10 mg bid the next day and maintained) + routine treatment	12	Pwd, effective rate
Wang AH 2011[27]	232(117/115)	t:60.1±10.1 c:63.0±9.0	48.71%	HBP CHD DM HF	SSYX capsule 1.6 g tid + propafenone (150 mg, tid) + routine treatment (specific drug was unclear)	Propafenone (150 mg, tid) + routine treatment	8	Frequency of PAF attack
Liu YX 2013[23]	111(52/59)	t: 75.4±16.1 c: 74.4±16.3	53.15%	CHD	SSYX capsule 1.6 g tid + amiodarone (0.2 g tid, decreased to bid a week later and decreased to qd the next week later and maintained at qd.) + routine treatment (specific drug was unclear)	Amiodarone (0.2 g tid, decreased to bid a week later and decreased to qd the next week later and maintained at qd.) + routine treatment	8	Pwd
Jin YW 2012[24]	45 (21/24)	t: 63.94±14.26 c: 62.74±12.74	57.78%	HBP RHD	SSYX capsule 1.2 g tid + metoprolol (23.75 mg qd) + routine treatment (specific drug was unclear)	Metoprolol (23.75 mg qd, increased to 47.5 mg qd according to tolerance capability of patients)+routine treatment	12	Pwd, CRP
Wu PT 2007[25]	112 (58/54)	t: 56±3 c: 59±4	57.14%	HBP CHD HF	SSYX capsule 1.6 g tid + metoprolol (dosage is unclear) + routine treatment (specific drug was unclear)	Metoprolol (dosage is unclear) + routine treatment	12	Pwd
Han YH 2007[28]	120 (60/60)	t: 58±16 c: 54±11	67.50%	HBP CHD RHD	SSYX capsule 1.6 g tid +metoprolol (dosage is unclear) + routine treatment (specific drug was unclear)	Metoprolol (dosage is unclear) + routine treatment	12	Frequency of PAF attack, Life quality
Cao D 2013[29]	116 (58/58)	t: 62.1±9.3 c: 61.3±9.7	59.48%	HF	SSYX capsule 0.8–1.6 g tid +antiarrhythmic drugs (unclear) + routine treatment (specific drug was unclear)	antiarrhythmic drugs (unclear) + routine treatment	20	Frequency of PAF attack, Pwd
Liu H 2011[30]	120 (60/60)	t: unclear c: 57±13	65.83%	HBP CHD RHD	SSYX capsule 1.6 g tid +antiarrhythmic drugs (unclear) + routine treatment (specific drug was unclear)	antiarrhythmic drugs (unclear) + routine treatment	12	Frequency of PAF attack, Life quality
Zhou FY 2008[31]	120 (60/60)	t: 58±16 c: 54±11	75.83%	HBP CHD RHD	SSYX capsule 1.6 g tid +antiarrhythmic drugs (unclear) + routine treatment (specific drug was unclear)	antiarrhythmic drugs (unclear) + routine treatment	12	Frequency of PAF attack
Tang YK 2012[32]	102 (51/51)	t: 76.3 c:75.7	44.12%	NR	SSYX capsule 0.8–1.6 g three to four times a day + antiarrhythmic drugs (unclear) + routine treatment (specific drug was unclear)	antiarrhythmic drugs (unclear) + routine treatment	8	Frequency of PAF attack, Maintaining sinus rhythm
Zhang CM 2012[33]	52 (26/26)	NR	53.85%	HBP CHD RHD	SSYX capsule 1.2 g tid + metoprolol 47.5 mg qd + routine treatment (specific drug was unclear)	Metoprolol 47.5 mg qd + routine treatment	24	Frequency of PAF attack, Life quality
Yu ZW 2010[34]	112 (58/54)	t: 55.2 c:52.6	59.82%	HBP CHD RHD	SSYX capsule 1.6 g tid + amiodarone (0.2 g tid, decreased to bid a week later and decreased to qd the next week later and maintained at qd.) + routine treatment (specific drug was unclear)	Amiodarone (0.2 g tid, decreased to bid a week later and decreased to qd the next week later and maintained at qd.) + routine treatment	12	Pwd
Li FC 2009[35]	72 (40/32)	NR	59.72%	NR	SSYX capsule 1.2 g tid + amiodarone (0.6 g qd, decreased to 0.4 g qd a week later and decreased to 0.2 g qd the next week later and maintained at 0.2 g qd.) + routine treatment (specific drug was unclear)	Amiodarone (0.6 g qd, decreased to 0.4 g qd a week later and decreased to 0.2 g qd the next week later and maintained at 0.2 g qd.) + routine treatment	24	Maintaining sinus rhythm, LAD
Ge KF 2009[36]	52 (26/26)	t: 42.5±4.5 c: 42.6±4.3	55.77%	HBP CHD RHD	SSYX capsule 1.6 g tid + amiodarone (0.2 g qd) + routine treatment (specific drug was unclear)	Amiodarone (0.2 g qd) + routine treatment	24	Maintaining sinus rhythm

For outcome measures, eight trials reported the Pwd, which was measured using surface ECG. Three trials reported maintenance of sinus rhythm and five trials reported quality of life, which was measured using the QOF scale. Twelve trials reported frequency of PAF attack. Only one trial reported improvement to PAF-related symptoms, such as palpitations, chest distress, short of breath and vertigo. Five trials reported adverse events.

### Methodological quality

**Fig 2:**
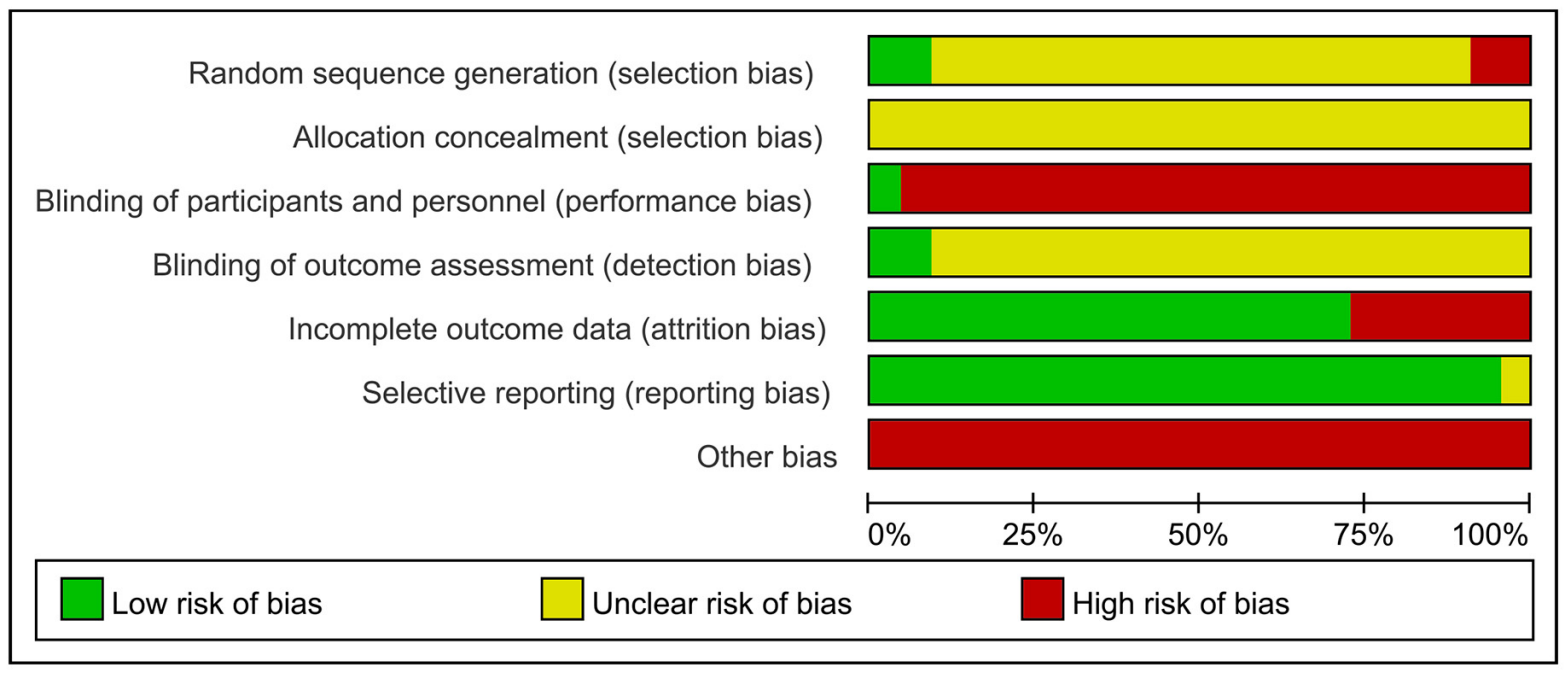
Risk of bias summary: review of the authors’ judgments regarding the risk of bias for each item for the included studies.

**Fig 3:**
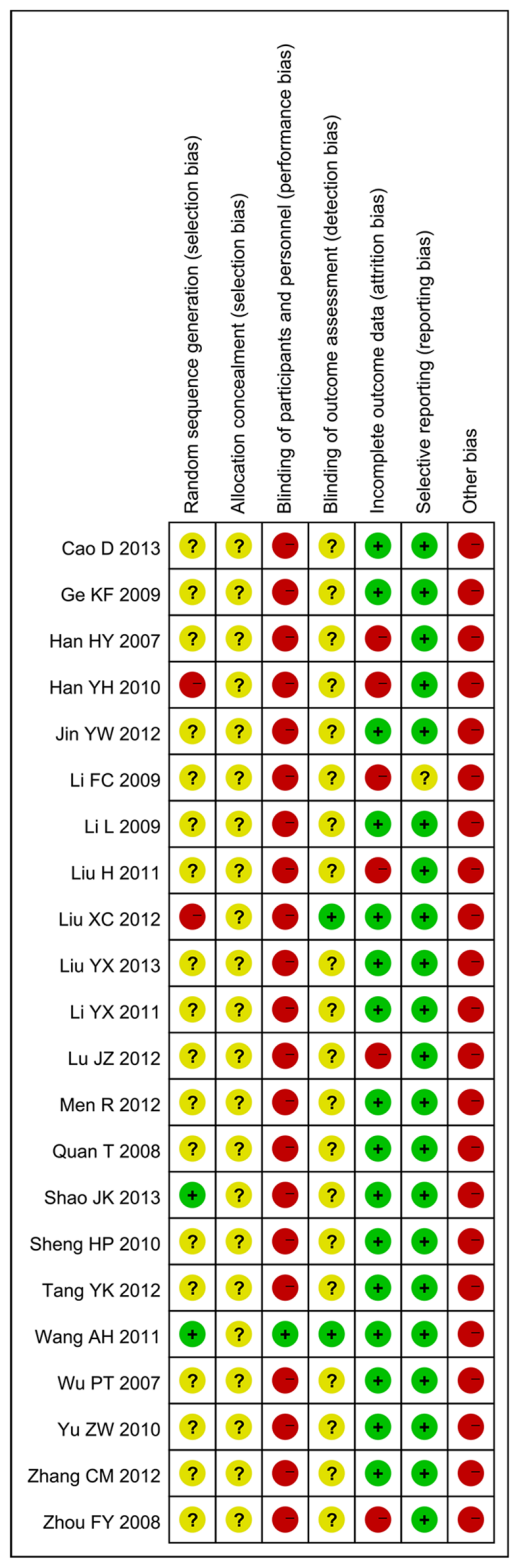
Risk of bias graph: review of the authors’ judgments regarding the risk of bias for each item, presented as percentages across all included studies.

All included trials were parallel-group randomized trials, but only two trials [15, 27] (9.09%, 2/22) reported the method of sequence generation (use of a random number table). No trial described allocation concealment. Only one trial mentioned blinding. Sixteen trials (72.72%, 16/22) reported on the drop-out of participants, but intention-to-treat analysis was not used. For selective reporting, because the protocols of the 22 trials were all not accessible, we made our judgments by comparing the outcome measures mentioned in the methods section with the reported outcomes in the results: 21 trials (95.45%, 21/22) that reported all outcome measures that were described in the methods were evaluated as low risk, and one trial [35] (4.54%, 1/22) was evaluated as unclear because no description of outcome measures was included in the methods. No trial provided a pretrial sample size estimation. All trials were evaluated as having a high or unclear risk of bias (Figs 2 and 3).

### Effects of the interventions

Three comparisons, including 6 trials that compared SSYX plus routine treatment to routine treatment alone, 8 trials that compared SSYX plus routine treatment to anti-arrhythmia drugs plus routine treatment, and 14 trials that compared SSYX combined with anti-arrhythmia drugs plus routine treatment to the same anti-arrhythmia drugs plus routine treatment, were identified. (Table 2).

**Table 2:** GRADE summary of 14 RCTs comparing SSYX combined with anti-arrhythmic drugs plus routine treatment to anti-arrhythmic drugs plus routine treatment in PAF patients. CI, Confidence interval; RR, Risk ratio.

Outcomes	Illustrative comparative risks (95% CI)		Relative	No of Participants	Quality of the evid
	Assumed risk: Anti-arrhythmia drugs plus routine treatment	Corresponding risk: SSYX combined with anti-arrhythmia drugs plus routine treatment	effect (95% CI)	(studies)	ence (GRADE)
Pwd	The mean pwd in the control groups was 40.95	The mean pwd in the intervention groups was 6.74 lower (9.34 to 4.14 lower)		537(6 studies)	⊕⊕⊕⊝moderate^a^
Quality of Life	The mean quality of life in the control groups was 31.79	The mean quality of life in the intervention groups was 25.98 higher (23.3 to 28.65 higher)		266(3 studies)	⊕⊕⊝⊝low^a^^,^^b^
Frequency of PAF attack/week	The mean frequency of paf attack/week in the control groups was 11.9	The mean frequency of paf attack/week in the intervention groups was 2.3 lower (5.34 lower to 0.75 higher)		602(6 studies)	⊕⊕⊕⊝moderate^a^
Maintenance of Sinus Rhythm	Study population: 583 per 1000; Moderate: 563 per 1000	Study population: 822 per 1000 ((688 to 986)); Moderate: 794 per 1000 ((664 to 951))	RR 1.41 (1.18 to 1.69)	224(3 studies)	⊕⊕⊝⊝low^b^

^a^ according to the ROB graph(Fig 3)

^b^ Total number of events is less than 300

#### P-wave dispersion

Eight trials used Pwd as an outcome measure. No significant difference was found in Pwd values before treatment between the experimental groups and control groups. This allowed a comparison of Pwd values after treatment.

**Fig 4:**
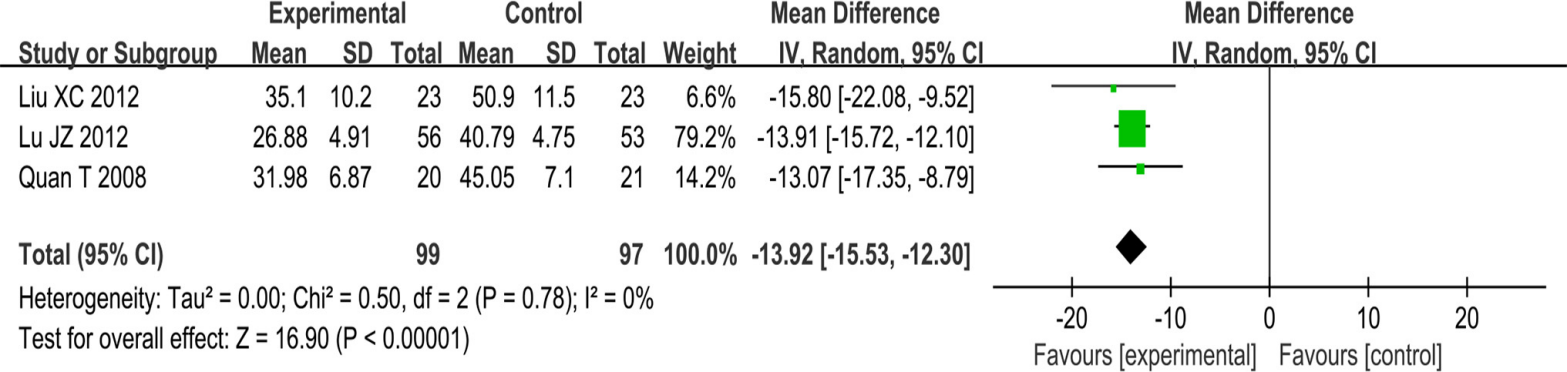
Forest plot of the comparison between SSYX plus routine treatment and routine treatment alone for the outcome Pwd.

SSYX plus routine treatment vs routine treatment. Evidence from a pooled analysis across three studies showed that Pwd after treatment with SSYX plus routine treatment was lower than Pwd after routine treatment alone. The meta-analysis demonstrated a significant difference between the two groups (MD:-13.92; 95% CI, -15.53 to -12.30; p<0.00001; I^2^ = 0%) (Fig 4).

SSYX plus routine treatment vs anti-arrhythmia drugs plus routine treatment. One trial [24] favored SSYX plus routine treatment for reducing Pwd, while the other three trials [20, 23, 25] reported that there was no significant difference between the two groups.

**Fig 5:**
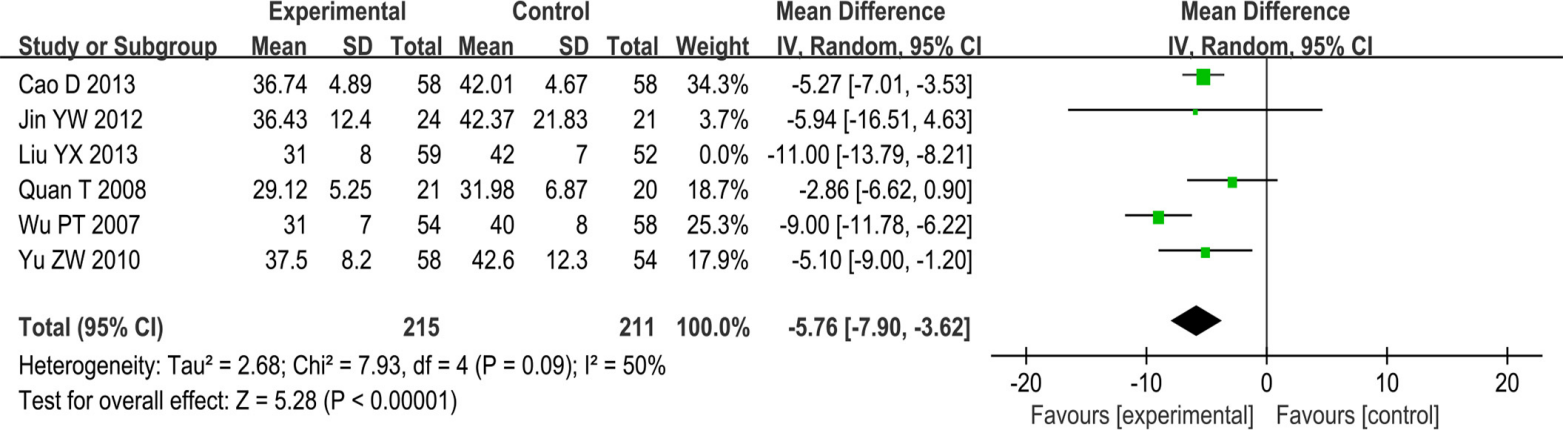
Forest plot of the comparison between SSYX combined with anti-arrhythmia drugs plus routine treatment and anti-arrhythmia drugs plus routine treatment for the outcome Pwd.

SSYX combined with anti-arrhythmia drugs plus routine treatment vs anti-arrhythmia drugs plus routine treatment. Six trials involving 537 patients showed heterogeneity (I^2^ = 74%) and therefore required the sensitivity analyses. We excluded one trial whose treatment duration was 8 weeks, while the treatment duration of the others was 12 weeks. The I^2^ decreased from 74% to 50%, which was acceptable and required a random effects model for statistical analysis. This meta-analysis demonstrated a difference between the two groups (MD-5.76; 95% CI, -7.90 to -3.62; p<0.00001; I^2^ = 50%) (Fig 5). We considered whether the heterogeneity might result from a peculiarity of the treatment duration.

#### Maintenance rate of sinus rhythm

**Fig 6:**
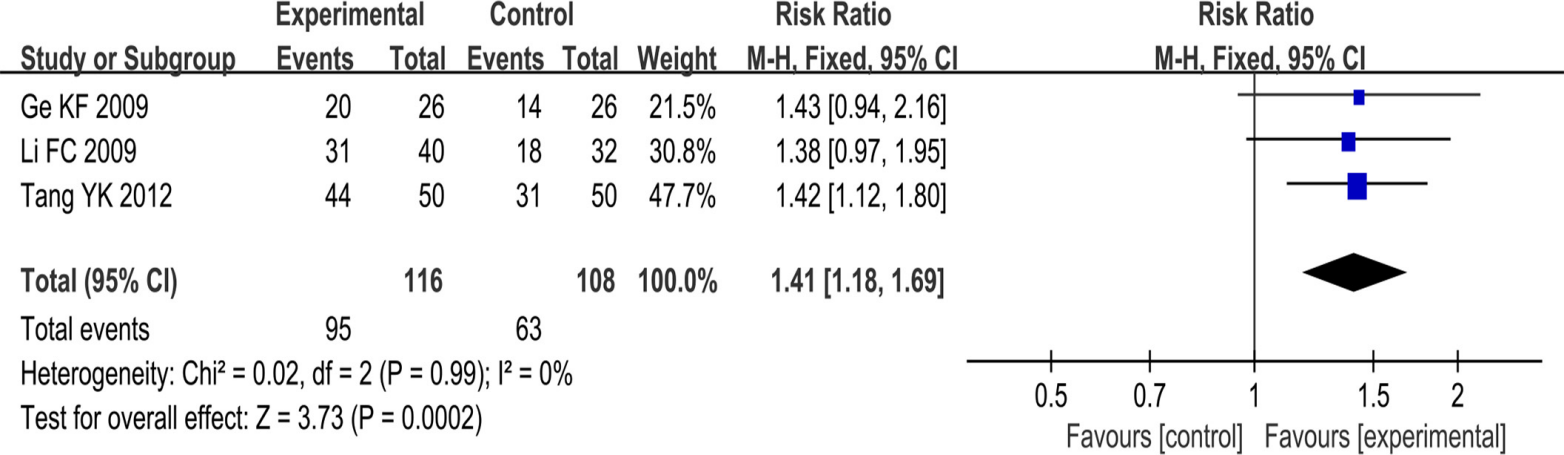
Forest plot of the comparison between SSYX combined with anti-arrhythmia drugs plus routine treatment and anti-arrhythmia drugs plus routine treatment for the outcome Maintenance of Sinus Rhythm.

Three trials used the maintenance rate of sinus rhythm as an outcome measure. These three trials compared the use of a combination of SSYX plus anti-arrhythmic drug and routine treatment to the use of an anti-arrhythmic drug plus routine treatment. The rate of maintenance of sinus rhythm in the SSYX combined with anti-arrhythmic drug group plus routine treatment was greater than that of anti-arrhythmic drug plus routine treatment group. The meta-analysis showed that there was a significant beneficial effect in the experimental group compared to the control group (RR: 1.41; 95% CI, 1.18 to 1.69; p<0.0002; I2 = 0%) (Fig 6).

#### Quality of life

**Fig 7:**
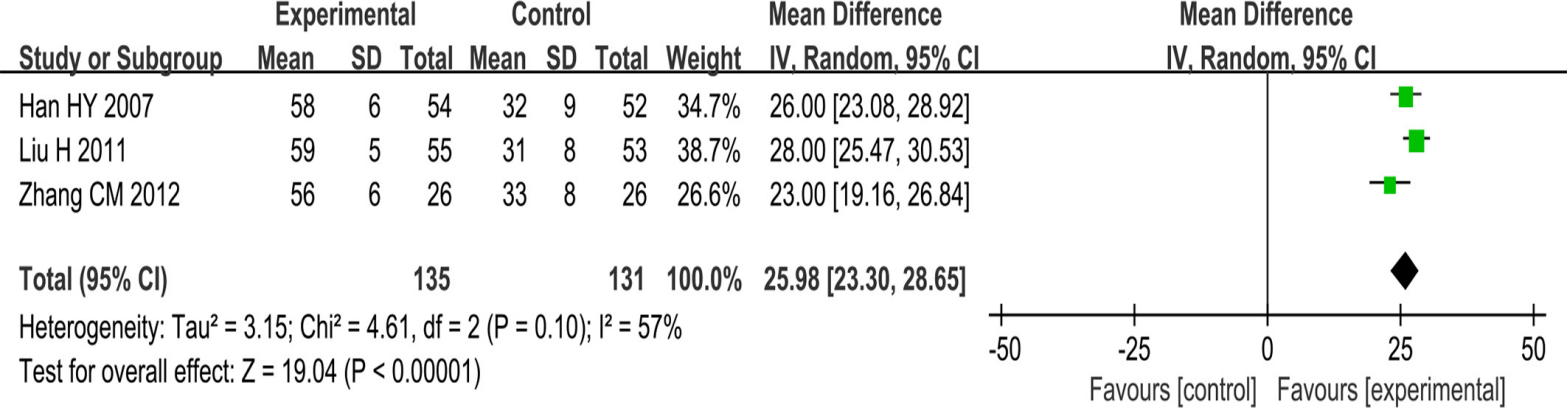
Forest plot of the comparison between SSYX combined with anti-arrhythmia drugs plus routine treatment and anti-arrhythmia drugs plus routine treatment for the outcome Quality of Life.

Five trials used the quality of life as an outcome measure. One trial [17] found that SSYX plus routine treatment was superior to routine treatment alone in improving QOL scores. One trial [21] favored SSYX plus routine treatment over anti-arrhythmic drug plus routine treatment with regard to QOL. A meta-analysis of three studies involving 266 patients showed that SSYX combined with anti-arrhythmia drugs plus routine treatment had a better effect on improving QOL scores than anti-arrhythmia drugs plus routine treatment and that there was a difference between the two groups (MD: 25.98; 95% CI, 23.30 to 28.65; p<0.00001; I2 = 57%) (Fig 7).

#### Frequency of PAF attack

Twelve trials used the frequency of PAF attack as an outcome measure. No significant difference in frequency of PAF attack before treatment was observed between the experimental groups and control groups, which allowed a comparison of the frequency after treatment.

**Fig 8:**
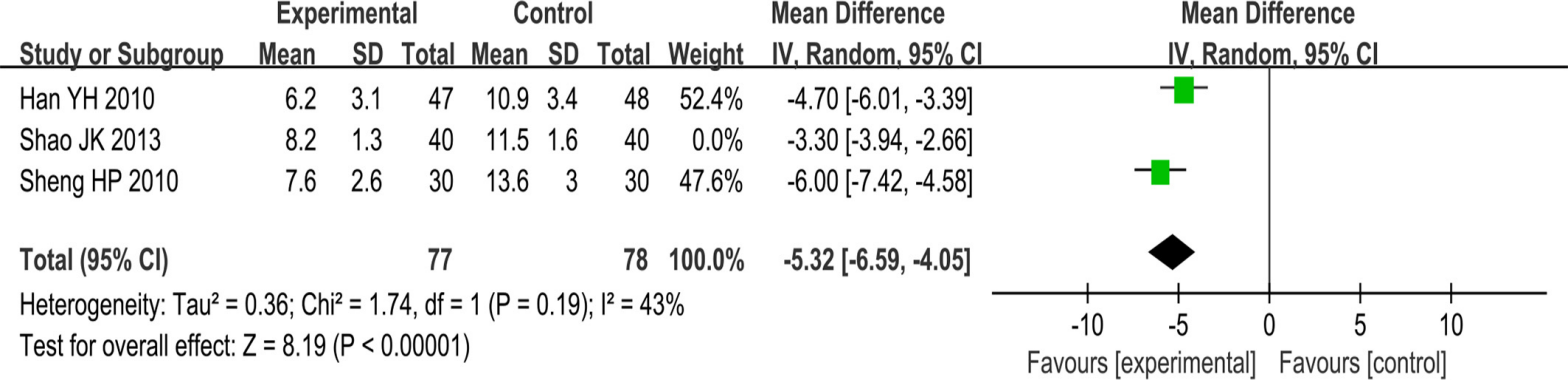
Forest plot of the comparison between SSYX plus routine treatment and routine treatment for the outcome frequency of PAF attack.

SSYX plus routine treatment vs routine treatment. Three studies involving 235 patients showed heterogeneity (I^2^ = 85%) and therefore required the sensitivity analyses. We excluded one trial whose dosage of SSYX was 1.2g tid and the treatment duration was 8 weeks, while others’ dosage was 1.6g tid and treatment duration was 24 weeks. Thus the I^2^ was reduced and a random effects model for statistical analysis was used. The meta-analysis demonstrated a difference between the two groups (MD:-5.32; 95% CI, -6.59 to -4.05; p<0.00001; I^2^ = 43%) (Fig 8).

SSYX plus routine treatment vs anti-arrhythmia drugs plus routine treatment. One trial [21] reported that there was no significant difference between SSYX plus routine treatment and anti-arrhythmic drugs plus routine treatment in reducing the frequency of PAF attack (MD: 0.07; 95% CI, -1.28 to 1.42; p>0.05). However, another trial [22] found that SSYX plus routine treatment was superior to anti-arrhythmic drugs plus routine treatment in reducing the frequency (MD: -5.40; 95% CI, -6.78 to -4.02; p<0.05). One trial [27] used quartiles as a parameter and showed that there was no difference between the two groups (p>0.05).

**Fig 9:**
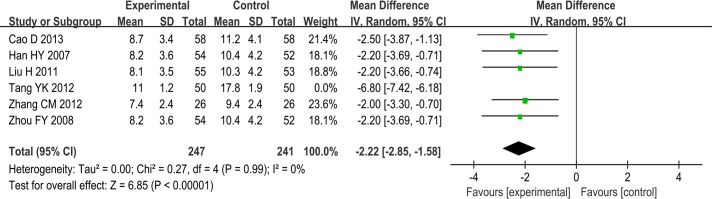
Forest plot of the comparison between SSYX combined with anti-arrhythmia drugs plus routine treatment and anti-arrhythmia drugs plus routine treatment for the outcome Frequency of PAF attacks per week (Tang YK 2012 excluded).

SSYX combined with anti-arrhythmia drugs plus routine treatment vs anti-arrhythmia drugs plus routine treatment. Even when a random-effects model was applied, heterogeneity was too large (I^2^ = 94%). This may have been due to clinical heterogeneity or to low methodological quality. We excluded one trial that included older patients [32], and the pooling data showed that SSYX combined with anti-arrhythmic drugs plus routine treatment had a better effect on reducing frequency of PAF attack than anti-arrhythmia drugs plus routine treatment (MD: -2.22; 95% CI, -2.85 to -1.58; p<0.00001; I^2^ = 0%). We therefore considered whether heterogeneity may have resulted from a peculiarity of some of the included participants (Fig 9). Another trial [27] used quartiles as a parameter and showed that there was no difference between the two groups (p>0.05).

#### Symptom improvement

Only one trial [26] reported symptom improvement. This trial compared SSYX plus routine treatment to anti-arrhythmic drug plus routine treatment and showed that SSYX plus routine treatment had a better effect on improving symptoms, such as palpitations, chest distress, shortness of breath, insomnia, drowsiness and vertigo, than anti-arrhythmic drugs plus routine treatment.

### Publication bias

We could not perform a funnel plot analysis to detect publication bias because there were an insufficient number of trials.

### Adverse effect

Only four trials (4/22, 18.18%) reported adverse events. In all, 16 of these incidents were reported in the treatment group, and 37 of these incidents were reported in the control group. The side effects included nausea, vomiting, gastrointestinal discomfort, stomach ache, constipation, chest distress, rash, thirst, impaired liver function, dizziness, palpitations, bradycardia, atrioventricular block and QT interval prolongation.

## Discussion

### Main findings

From our results, SSYX appears to be a relatively effective and safe treatment option for the treatment of PAF, and SSYX combined with anti-arrhythmic drugs plus routine treatment appears to be more effective than anti-arrhythmic drug plus routine treatment. The meta-analysis demonstrates that SSYX plus routine treatment was more effective at treating Pwd and the frequency of PAF attack compared with routine treatment alone (including aspirin and statins) and that SSYX combined with anti-arrhythmic drugs plus routine treatment conferred advantages in outcomes including Pwd, quality of life, frequency of PAF attack and maintenance rate of sinus rhythm in patients with PAF. However, the results from the included trials comparing SSYX plus routine treatment and arrhythmic drugs plus routine treatment were inconsistent. We found that one of four trials [24] favored SSYX plus routine treatment over anti-arrhythmic drugs plus routine treatment with regards to Pwd, that one of three trials [22] favored SSYX plus routine treatment with regards to the frequency of PAF attacks and that one trial [26] favored SSYX plus routine treatment for improving symptoms, such as palpitations and chest distress.

It has been shown that TCM is a holistic system of medicine that has unique theories and methods for its treatments, especially for relieving symptoms [37–38]. Compared to conventional anti-arrhythmic drugs, SSYX has the potential to be more effective for the improvement of clinical symptoms. It is widely known that the side effects of most conventional anti-arrhythmic drugs can lead to arrhythmia, and SSYX might offset some of these adverse events when used in combination with conventional anti-arrhythmic drugs.

### Strengths and limitation

The strength of our study is that it includes a large number of relevant evidence and presents a relatively rigorous illustration of the findings. Moreover, we have published the protocol for this study in PROSPERO.

This review also has its limitations. The methodological quality of the included trials was not promising. The majority of the trials did not provide sufficient information of randomization procedures or for proper concealment and blinding. Although we endeavored to contact the trial authors to clarify the missing information, responses were not satisfactory. Therefore, the placebo effect can not be ruled out in the current study. Moreover, intention-to-treat analyses and pre-trial sample size estimates were not applied in all trials. We could not perform a funnel plot analysis because there was an insufficient number of included trials in the meta-analysis. Therefore, potential publication bias can not be ruled out in this study. Because all of the trials were conducted in China, geographic biases may be present. We did not find evidence for the long-term effectiveness of SSYX, which is limited mostly by the absence of follow-up studies. We did not perform a GRADE Profile to summarize all of the findings for outcomes because the power to pool all of the included trials was too low. Instead, we presented a GRADE summary of the trials that compared SSYX combined with anti-arrhythmic drugs plus routine treatment to anti-arrhythmic drugs plus routine treatment.

### Generalizability and applicability of results

Although this review shows that there appears to be some benefit from the use of SSYX, the results should be interpreted with caution due to the poor methodological quality of the included studies and potential clinical heterogeneity. Furthermore, the safety of using SSYX still needs to be shown.

## Conclusions

### Implications for practice

Due to the poor methodological quality and the limited number of eligible trials, we could not draw confirmative conclusions regarding the beneficial effect of SSYX. It should also be noted that SSYX should be used with caution, both when used alone and when combined with conventional arrhythmic drugs.

### Implications for further study

In the future, results from well-designed multicenter clinical trials with large sample sizes from studies following the CONSORT criteria for reporting sufficiently and clinically validated outcomes, especially double blind and double simulation clinical trials, are needed to draw more definitive conclusions regarding the effectiveness of SSYX and to rule out placebo effects.

Pwd could be a good outcome for reporting on PAF because tests for it are easier to perform and more accurate. Clinically relevant outcomes, such as QOL, should be addressed and measured using validated instruments. In addition, composite outcomes are never preferred, and we recommend that each outcome be reported individually with clear definitions and criteria.

## Supporting Information

S1 PRISMA Checklist PRISMA Checklist. (DOCX)
